# The Complex Gene–Carbohydrate Interaction in Type 2 Diabetes: Between Current Knowledge and Future Perspectives

**DOI:** 10.3390/nu17142350

**Published:** 2025-07-17

**Authors:** Francesca Gorini, Alessandro Tonacci

**Affiliations:** Institute of Clinical Physiology, National Research Council, 56124 Pisa, Italy; alessandro.tonacci@cnr.it

**Keywords:** type 2 diabetes, carbohydrates, whole grains, fiber, diet, nutrigenetics, nutrigenomics, epigenetics

## Abstract

Type 2 diabetes (T2D) represents a public health problem globally, with the highest prevalence reported among older adults. While an interplay of various determinants including genetic, epigenetic, environmental factors and unhealthy lifestyle, particularly diet, has been established to contribute to T2D development, emerging evidence supports the role of interactions between nutrients or dietary patterns and genes in the pathogenesis of this metabolic disorder. The amount, and especially the type of carbohydrates, in particular, have been correlated with the risk of non-communicable chronic disease and mortality. This narrative review aims to discuss the updated data on the complex and not fully elucidated relationship between carbohydrate–gene interactions and incidence of T2D, identifying the most susceptible genes able to modulate the dual association between carbohydrate intake and risk of developing T2D. The identification of genetic polymorphisms in response to this macronutrient represents a potentially powerful target to estimate individual risk and prevent the development of T2D in the context of personalized medicine. The postulation around novel foods potentially tailored to minimize the risks of developing T2D will pave the way for a new era into food research in relation to the safeguarding of well-being status in patients affected by, or at risk for, T2D.

## 1. Introduction

Diabetes, a chronic and serious condition resulting from the lack of production or inefficient use of insulin, is a major cause of morbidity and mortality and one of the fastest growing global health emergencies of the 21st century [1,2,3]. Among the different types of diabetes, type 2 diabetes (T2D) is characterized by hyperglycemia due to the progressive loss of adequate insulin production by pancreatic beta cells and it frequently occurs in the setting of insulin resistance in several tissues (e.g., adipose tissue, liver, skeletal muscles) and metabolic syndrome (MetS) [4]. T2D accounts for over 90% of diabetes cases worldwide, with an estimated prevalence of 462 million individuals affected (corresponding to 6.28% of the world population) and over 1 million deaths in 2017, making this condition the ninth leading cause of mortality [5,6]. The 2021 IDF Diabetes Atlas, which periodically produces prevalence estimates and future projections for diabetes, predicted that 643 million people (11.3% of the global population) will have diabetes by 2030 and 783 million people (12.2%) by 2045 [3]. A recent pooled analysis of 1108 population-representative studies, with a total of 141 million participants, reported a global age-standardized diabetes prevalence of 13.9% for women and 14.3% for men, with an estimated 828 million adult individuals with diabetes in 2022, an overall global increase of 630 million from 1990 [7]. In particular, the largest increases were recorded in low- and middle-income countries due to the almost total lack of treatment and inadequate primary prevention programs [7], highlighting the importance of disease prevention and personalization of care within a “p4 medicine” framework. The hyperglycemia in T2D patients can induce damage to various organs and tissues, in particular the kidneys, eyes, nerves, and circulatory system, leading to an increased risk of kidney failure, vision loss, cardiovascular, cerebrovascular, and other peripheral vascular diseases, and a two-fold excess risk of death from any cause compared to controls [6,8,9,10].

Overall, T2D is a complex disorder resulting from the interaction of anthropological factors including age, small or large birth weight, body mass index, and environmental factors such as sedentary lifestyle, high-calorie diets, obesity, environmental pollutants with genetic component, epigenetic modifications, gut microbiota, and organelle stress [11,12,13]. Indeed, although T2D is characterized by a strong genetic basis, with inheritance estimated between 20% and 80%, the approximately 700 genetic variants identified by genome-wide association studies (GWAS), half of which were discovered in the past three years, related to beta cell function, insulin secretion, and insulin resistance, account for almost 20% of the hereditability of the disease, clearly indicating the substantial contribution of other determinants to the disease’s development [12,13,14,15]. In particular, over the last two decades, gene–diet and dietary pattern interactions have emerged as key players in the pathophysiology of T2D. Nutrigenomics (the investigation of the effects of diet on gene expression) and nutrigenetics (the study of the impact of genetic variation on biological responses, particularly metabolic status, to dietary intake), both fields linked to advances in omics technologies, have been identified as research foundations for both personalized nutrition and precision healthcare for chronic non-communicable diseases such as T2D [12,16,17,18]. Prospective studies have provided suggestive insights into gene–diet interactions in relation to T2D, in the setting of the general pattern of gene–environment interactions, resulting in a synergistic or antagonistic effect on outcome that is greater or less than the sum or product of individual exposures [19,20]. At the same time, the high adherence to a healthy diet or, conversely, to a dietary pattern characterized by low amounts of antioxidants may reduce or increase the risk of T2D, respectively [21,22]. Furthermore, nutritional epigenetics, i.e., the study of molecular processes modulating gene expression affecting the genome sequence, induced by bioactive dietary components, represents a further novel field of study of the impact of nutrition on the risk of T2D and can potentially explain an individual’s susceptibility to develop the disease [23,24].

Carbohydrates, the most abundant molecules in nature and key sources for energy production and signaling pathways, have been associated with both a reduced and increased risk of T2D although high levels of intake have been recognized as closely related to the development of chronic metabolic diseases [25,26,27]. In fact, non-digestible forms of carbohydrates contain fiber, with beneficial effects on overall metabolic health including insulin sensitivity; on the other hand, an excess of mono- and disaccharides, typical of Western diets, shortens lifespan, contributing to an increased risk of chronic diseases such as obesity, T2D, metabolic syndrome, and non-alcoholic fatty liver disease [25,28].

Therefore, this critical review aimed to summarize the up-to-date evidence on the associations between carbohydrate intake and T2D risk, discuss the strengths and limitations of findings on the complex interaction between carbohydrate intake and the major genetic determinants in T2D and how epigenetic alterations may potentially modulate these relationships, and also propose future interventions such as the use of functional foods as a prevention and treatment strategy for T2D.

## 2. Genetics of Type 2 Diabetes

While the risk of T2D has been established to depend on both genetic and environmental causes, knowledge on the genetic basis for T2D is still incomplete [29,30]. Furthermore, unlike type 1 diabetes, the genetic risk of T2D is not concentrated in a single region and is otherwise the result of interactions between multiple genes scattered throughout the genome [11]. Based on large population-based studies, heritability (the degree to which genetic differences contribute to the number of phenotypic variations in a trait or disease) for T2D has been estimated at 20–80%, with great variability between populations possibly due to differences in age ranges, regions and ethnicities [30,31]. In particular, the risk of a child developing T2D in the case of an affected parent increases by 3 times compared to the general population and up to 6 times if both parents are affected by T2D, compared to subjects without a positive family history of the disease [32,33]. Likewise, a singleton sibling of an affected sibling has an approximately 3-fold increased risk of T2D, and the risk increased to more than 30-fold when the affected first-degree relatives were two or more siblings and one parent [34]. Higher concordance rates in monozygotic than in dizygotic twins further support the significant genetic component in T2D [35]. Conversely, no significant difference in genetic risk between males and females has been observed recently [30].

Over the last three decades, advances in sequencing and genotyping techniques have paved the way to linkage analyses (the primary method used in the second half of the 20th century to map genetic loci with familial aggregation [36,37]), which have allowed for the identification of the gene encoding the transcription factor 7-like 2 (*TCF7L2*), considered to be the most potent locus for T2D risk [38]. Subsequent candidate gene studies (based on an approach assessing the association between an allele or a group of alleles of a gene potentially involved in a disease and the disease itself [39]) then led to the hypothesis that the peroxisome proliferator-activated receptor gamma (*PPAR-γ*) may represent a predisposing factor for obesity and insulin resistance [38,40,41]. Although relatively successful in identifying rare variants in single-gene disorders, linkage analyses are less effective in detecting genes that are involved in polygenic disease such as T2D [11]. On the other hand, most genes identified in candidate gene studies, despite their involvement in glucose and lipid metabolism and insulin secretion and signaling, do not appear to be associated with T2D [11]. The advent of GWAS has made it possible to genotype hundreds of thousands to millions of markers, generally single-nucleotide polymorphisms (SNPs, the most common type of genetic variations between individuals), across the human genome to identify the role of common variants (those having a frequency ≥ 0.05) in the development of a trait or a disease [42]. Thanks to GWAS, more than 700 novel risk loci have been discovered, all accounting for an increased risk of T2D of less than 40%, and many of them of for less than 15% [39]. However, despite the large collection of loci reaching the conventional threshold of genome-wide significance (*p*  <  5  ×  10^−8^) [43], only a limited number of them have been explored for their interaction with carbohydrate intake and their subsequent impact on the development of T2D.

The product of the *TCF7L2* gene, located on chromosome 10, is a downstream effector involved in the canonical Wnt/β-caternin signaling cascade, which, in addition to being critical for pancreatic islet development and the production and secretion of insulin, has also been related to various common diseases as well as to diverse models of cancers [37,44,45]. SNPs of the *TCF7L2* gene, especially the rs7903146 (C/T) SNP, one of the most susceptible genes to T2D discovered so far, have been associated with almost 20% of T2D patients and exert their effects via a multiplicative genetic model [44,46]. A recent meta-analysis including 28 studies with a total of 56,628 participants (34,232 cases and 22,396 controls) reported a significant, strong association of rs7903146 with T2D in Caucasian, East Asian, South Asian, and other ethnicities, with an increased risk ranging from 41 to 81% [47]. In non-diabetic individuals, *TCF7L2* rs7903146 appears to be associated with a decrease in basal and glucose-stimulated insulin secretion as well as with quantitative and qualitative morphological changes in pancreatic islets [48]. The same variant is responsible for decreased β-cell responsivity and impaired glucose tolerance in obese adolescents [49]. TCF7L2, highly expressed in white adipose tissue, also has a fundamental role in adipogenesis by directly regulating the expression of genes implicated in lipid and glucose metabolism, while the loss of TCF7L2 in adipocytes leads to reduced glucose tolerance, increased insulin resistance, weight gain, and increased amount of subcutaneous adipose tissue in mice fed with high-fat diet, predisposing animals to develop T2D [40,50]. Furthermore, TCF7L2 suppression affects the normal function of pancreatic β-cells, resulting in a reduced glucose-stimulated insulin secretion, underlying defective insulin exocytosis [51] (Table 1).

PPAR-γ, highly expressed in adipose tissue, is a ligand-activated nuclear receptor that controls the transcriptional status of various genes involved in different biological functions such as adipocyte differentiation and immunity, cell differentiation, and glucose and lipid metabolism [52]. Importantly, thiazolidinediones, PPAR-γ agonists currently used as therapeutic agents in T2D subjects, primarily act in the adipose tissue where PPAR-γ is predominantly expressed, promote lipid uptake and storage, and stimulate lipogenic activities in fat cells, thereby leading to an improvement in insulin resistance and a reduction in blood glucose levels [53,54]. The *PPAR-γ2* rs1801282 missense variant, also known as Pro12Ala due to the replacement of proline with alanine, has been extensively examined in epidemiological studies, which generally reported a decreased risk of T2D associated with this variant [55,56,57]. In contrast, past research supported an association of *PPAR-γ2* rs1801282 with insulin resistance in both non-diabetic children and adults, which can also be modified by circulating lipids [57,58]. These conflicting results can be attributed to differences in populations, ethnicities, individual characteristics, and pre-existing conditions such as obesity or T2D [59]. Actually, a recent study showed that *PPAR-γ2* rs1801282 and *PPAR-β/δ* rs2016520—the latter a variant associated with PPAR-β/δ, another isoform belonging to the nuclear receptor superfamily, which appears to improve glucose and fatty acid metabolism and alleviate insulin resistance, glycogen storage, and gluconeogenesis downregulation in animal models—are associated with higher values of waist circumference, fasting plasma glucose, and glycosylated hemoglobin A1c (HbA1c, i.e., a relevant indicator reflecting the cumulative glycemic history during the previous two or three months) [60,61,62,63] (Table 1).

Glucose dependent insulin polypeptide receptor (*GIPR*) is another gene with a crucial role in metabolic pathways [64]. The *GIPR* gene, located on chromosome 19 in the β cells of Langerhans islands and, to a lesser extent, in insulin-sensitive tissues such as adipose tissue, encodes a G-protein coupled receptor for the GIP hormone [64,65]. GIP is an intestinal hormone secreted from the enteroendocrine K cells in the postprandial state, and it is responsible for the increased uptake of lipids and glucose, stimulation of insulin secretion, and inhibition of glucagon secretion from pancreatic cells in healthy subjects, although the incretin effect remains active 1–2 min after its secretion [66,67]. GIP also increases glucagon levels during fasting and hypoglycemic conditions, with little or no effect concomitant on insulin secretion, indicating a bidirectional function of this hormone in stabilizing glucose levels with opposite effects on the two main pancreatic glucoregulatory hormones [68]. Furthermore, in combination with hyperinsulinemia, GIP increases subcutaneous abdominal adipose tissue blood flow (ATBF) and stimulates triacylglyceride deposition in adipose tissue in lean humans without inducing any significant changes in ATBF response in obese subjects with decreased glucose tolerance [69,70]. *GIPR* rs10423928, due to T/A exchange, is associated with increased fasting glucose and proinsulin levels, and reduced ß-cell function, thereby representing a risk factor in non-diabetic individuals [71], while in subjects with T2D it causes a reduced incretin effect (no effect on insulin secretion) and consequent postprandial hyperglycemia [72,73] (Table 1).

Insulin receptor substrate ( ) genes encode a family of cytoplasmic adaptor proteins that are phosphorylated by the intrinsic tyrosine kinase activity of the insulin receptor, which is activated upon binding of the hormone and then in turn activates the downstream phosphatidylinositol 3-kinase pathway (PI3K), mediating key actions of insulin [74,75]. IRS molecules (i.e., IRS-1, IRS-2, IRS-3, IRS-4, which show differences in tissue and subcellular localization, phosphorylation patterns, developmental expression, and binding to the insulin receptor) represent pivotal mediators in insulin signaling pathways, which play a central role in maintaining essential cell functions such as growth, metabolism, and survival [76]. Although several SNPs have been identified in *IRS1* genes, only the Gly to Arg972 substitution of *IRS-1* (rs1801278 SNP) seems to represent a key independent determinant in the onset of insulin resistance in T2D patients, affecting the ability of insulin to activate the PI3K cascade that physiologically regulates glucose metabolism by promoting glycolysis and inhibiting gluconeogenesis in insulin-sensitive tissues [77,78,79]. However, as previously observed for *PPAR-γ*, the effects of *IRS-1* may vary depending on ethnicity, presence of obesity, and sample size [77]; therefore, studies conducted on certain Asian populations did not find any association of *IRS-1* with insulin resistance and/or risk of T2D [79,80,81] (Table 1).

Meta-analyses of GWAS datasets, which increase the power of association signals by increasing sample size through the assessment of multiple datasets [82], have allowed for the identification of hundreds of T2D susceptibility loci up to 2023, based on the finding that the frequency of a specific SNP is higher in cases than in controls and thus associated with the disease; however, they explain only a small effect on the heritability of T2D [83,84]. Therefore, the “missing heritability” in T2D could be attributed to the presence of common variants with a lower frequency (≥1%), those not yet identified, and/or rare variants detectable only by whole-genome sequencing (WGS) or whole-exome sequencing (WES), which, while allowing for assessment of the full spectrum of genetic variation, are based on a small sample size [37,85,86,87]. Rare variants in single genes are the cause of monogenic diabetes (MD), a form of diabetes with autosomal dominant inheritance, frequently misdiagnosed as type 1 diabetes or T2D, and representing approximately 2.5–6% of all diabetes cases [88,89]. In particular, maturity-onset diabetes of the young (MODY) is the most common form of MD and, to date, at least 14 different genes have been associated with MODY development [89]. Furthermore, WGS data have recently shown that rare variants may explain a large proportion of the heritability of complex traits and diseases, including T2D, for which rare variants are responsible for up to 25% of the heritability of the strongest common single-variant signals [86,90].

**Table 1:** Characteristics of the most relevant loci possibly involved in pathogenesis of type 2 diabetes.

Gene	Acronym	Original Function	Variant	Role in T2D	References
Transcription factor 7-like	*TCF7L2*	Encoding a Wnt signaling-associated transcription factor	rs7903146	Decrease in insulin secretion; morphological and functional changes in β cells	[48,49,50,51]
Proliferator-activated receptor gamma	*PPAR-γ*	Encoding a ligand-activated superfamily member of ligand-dependent transcription	rs1801282	Increase in insulin resistance; impairment of anthropometric, glucose, and lipid metabolism biomarkers	[58,59,60]
Glucose dependent insulin polypeptide receptor	*GIPR*	Encoding a G protein-coupled receptor for the GIP hormone	rs10423928	Increased fasting glucose and proinsulin levels; reduced incretin effects	[69,70]
Insulin receptor substrate-1	*IRS-1*	Encoding a cytoplasmic adaptor protein involved in insulin signal transmission	rs1801278	Increased insulin resistance	[77,78,79]

## 3. Carbohydrate–Gene Interactions in Type 2 Diabetes

The interest towards the importance of diet as a modifiable risk factor in T2D has been progressively increasing in recent decades, with most evidence resulting from a multitude of cross-sectional and cohort studies published since the late 1990s [15,91]. On the other hand, according to the “developmental-origins hypothesis”, perinatal events (prematurity, low birth weight), maternal nutrition imbalance, and medication intake during pregnancy, which lead changes in metabolism and hormone production that determine alterations in the development and functioning of various organs, may profoundly affect subsequently adulthood susceptibility to certain chronic diseases such as T2D, MetS, coronary heart disease, obesity, and stroke [92].

More recently, a number of systematic reviews and meta-analyses have assessed the relationship between food and dietary factors and the onset of T2D [93]. This condition arises from a complex interaction between genetic and lifestyle factors, with diet quality likely to play a key role in the causal mechanisms of T2D development, although the generally poor quality of past studies due to cross-sectional design (subject to recall bias), small sample sizes, and inadequate control for confounders, has produced unconclusive evidence [19,94,95]. As suggested by Franks et al., large case–control studies nested with population-based cohorts, assessing interactions selected on a priori biologically driven-hypotheses to reduce the likelihood of spurious results, would instead be desirable to provide useful information in the investigation of disease etiology [94]. Actually, larger prospective studies have been conducted since then, and novel findings in the field of diet-gene interactions have recently been published [19,95]. In the following sections, we will focus on the bidirectional role of carbohydrates in the occurrence of T2D and how they can interact with susceptibility genes.

### 3.1. Carbohydrates and Their Role in Human Health

**Figure 1:**
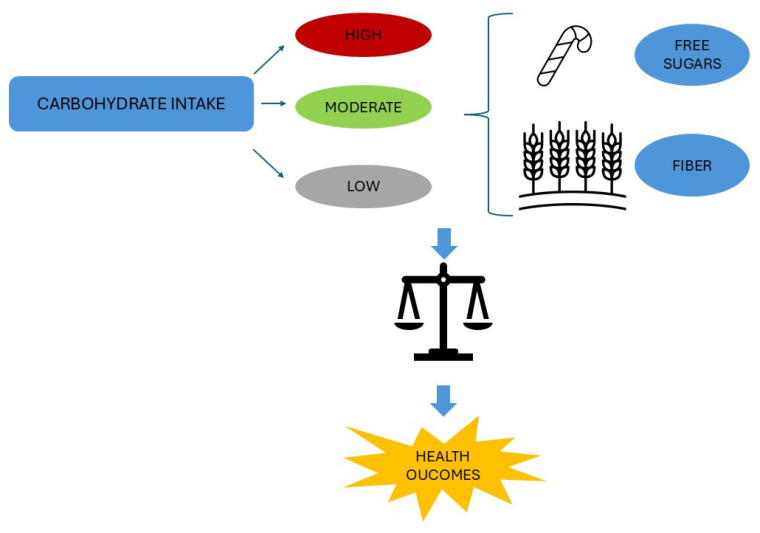
Schematic representation of the trade-off between quantity and quality of carbohydrate consumption and the risk of human disease.

Along with proteins and fats, carbohydrates are also known as macronutrients that provide essential energy for the maintenance of biological systems [25]. Carbohydrates can be divided into two main groups: simple, consisting of one (i.e., monosaccharides such as fructose, galactose, glucose) or two sugar units (i.e., disaccharides like lactose, maltose, sucrose); and complex, which are composed of many sugar units including starch, glycogen, and fibers [95]. Cereals, especially in the form of whole grains (WGs), a category of foods consisting of endosperm, germ, and bran, represent a relevant source of fibers known as non-digestible carbohydrates and composed of at least 10 monomers [28,96]. Based on the 2020–2025 Dietary Guidelines for Americans, the reference intake of carbohydrates corresponds to 45–65% of total daily calories [97]. Notably, both low (<40%) and high carbohydrate (>70%) intakes have been related to an increase in mortality compared to a moderate intake (50–55%) [98]. Indeed, low-carbohydrate diets may result in accumulating animal fat and protein with a concomitant reduction in vegetable protein and dietary fiber intake, which overall are significantly associated with T2D risk and increased mortality from cardiovascular disease (CVD) [99,100,101]. Dietary fiber, classified as soluble or insoluble depending on its solubility in water, reduces the aging process and therefore the risk of mortality, especially at high intakes [25,28]. Dietary fiber presents further beneficial effects, promoting healthy gut microbiota, increasing the feeling of satiety and thereby facilitating weight loss, and regulating carbohydrate and lipid metabolism [25,28]. Consistently, dietary patterns such as the Mediterranean and Okinawan diets, both characterized by a protein/carbohydrate ratio of less than 1, are associated with reduced overall mortality, lower risk of major chronic and metabolic diseases, and increased life expectancy [28,102]. In addition to the daily amount of carbohydrates, carbohydrate type appears to be crucial for health outcomes [25]. In fact, the inverse association between adherence to the Mediterranean diet and mortality is attributable both to moderate carbohydrate consumption and to a dietary pattern mainly composed of high fiber from vegetables and fruits and WG products as the main sources of carbohydrates [103,104]. Given the health benefits of WG consumption due to their high fiber and antioxidant and phytochemical contents, current U.S. Dietary Guidelines recommend that WGs make up at least 50% of total grain intake, with the remainder coming from refined grains that have had the bran removed during the milling process and whose consumption has been associated with an increased risk of metabolic disease [26,95,96,105]. However, as is generally the case with other foods, WGs are not generally consumed alone but combined with other food groups or macronutrients [106]. Intake of food items rich in natural (those contained in fruits and vegetables) and added sugars (those contained in sweetened beverages, fruit juice, preserves, and added during processing or preparation) has been associated with all-cause, cardiovascular, and cancer mortality, with sugar intakes > 20% of total energy linked to a 30% increase in the risk of mortality [25,107,108,109]. Given the threat to human health associated with an excessive consumption of free sugars, which may cause de novo lipogenesis, inflammation, and oxidative stress, the World Health Organization, beyond recommending an intake of free sugars at less than 10% of the total energy intake, suggests a further reduction to less than 5% [110]. Therefore, there is a fine balance underlying the relationship between carbohydrate intake, the quality of carbohydrates ingested, and the risk of diseases, including T2D, with opposite effects (Figure 1).

### 3.2. Whole-Grain Intake and Risk of Type 2 Diabetes

A series of meta-analyses have assessed the relationship between WG intake and other types of grains, namely refined grains, and the risk of T2D, with results suggesting significant associations, although with limitations that reduce their strength and evidence (Table 2). A meta-analysis by Ye et al., including a total of 45 prospective cohort studies and 21 randomized-controlled trials (RCTs) published in the years 1966–2012, reported that WG intake was associated with a 26% reduced risk of T2D (relative risk—RR = 0.74, 95% confidence interval—95% CI: 0.69–0.80) [111]. This estimate was based on six observational studies (for a total of 288,410 participants) comparing the highest intake level (consumers of an average of 48–80 g/day—3–5 serving/day of whole grains) with the lowest one (rare or never consumers of whole grains) [111]. Furthermore, based on 11 prospective studies and 389,319 subjects, the risk of T2D related to the comparison between the highest and the lowest category of total cereal fiber intake was reduced by 13% (RR = 0.87, 95% CI: 0.81–0.94) [110]. A subsequent meta-analysis encompassing 16 cohort studies (ten of which assessed the relationship between WG and T2D and included 19,829 cases among 385,868 participants, and six studies examining the impact of refined grains for a total of 9545 cases among 258,078 participants) published up to 2013 found a 32% reduced risk for T2D associated with three servings a day of WGs (RR = 0.68, 95% CI: 0.58–0.81) [96]. The authors observed a nonlinear relationship showing most of the risk reduction with an increasing intake up to two servings a day, but no association between refined grain intake and occurrence of T2D (RR = 0.95, 95% CI: 0.77–1.04) [96]. Similarly, a significantly inverse association was found in subgroup analyses for WG bread, WG cereals, wheat germ, and brown rice with T2D risk, while T2D risk had a positive association with white rice [96]. Although based on an extremely small amount of data, these results can be explained by differences in WGs in their content of fiber, antioxidants, phytochemicals, and micronutrients, which subsequently influence cardiovascular health [96,112,113,114]. The meta-analysis by Chanson-Rolle and co-authors [106], including eight observational studies (all but one were prospective, with a follow-up interval of 6 to 22 years) comprising 15,573 cases among 316,051 participants, corroborated previous findings and reported a significant inverse association between WG intake and the incidence of T2D, with a 0.6% decrease in T2D risk for each 20 g/day increase in WG consumption, and the relationship remained significant even after adjusting for covariates. Using data from the Danish Diet, Cancer, and Health cohort including 55,465 participants of whom 7417 received a diagnosis of T2D during the follow-up, Kyrø et al. [115] investigated the relationship between different WG intakes and risk of T2D. Each one-serving increase in WG intake (16 g daily) was associated with a significantly reduced risk of T2D by 11% (hazard ratio—HR = 0.89, 95% CI: 0.87–0.91) in men and by 7% in women (HR = 0.93, 95% CI: 0.91–0.96) [115]. Likewise, a one-serving increment (50 g/day) in WG product intake was also related to a significantly lower risk of T2D in both sexes (HR = 0.89, 95% CI: 0.86–0.90 in men and HR = 0.93, 95% CI: 0.90–0.96 in women) [115]. When examining the impact of three WGs (wheat, rye and oats), they were all significantly associated with a reduced risk of T2D from 34 to 8% in men, while in women wheat alone showed a significant inverse association (by 21%) [115]. Reynolds et al. [116], who conducted a series of systematic reviews and meta-analyses of prospective studies (published by 2017) and RCTs (published by 2018) to assess the correlation between carbohydrate quality and selected health outcomes, demonstrated that a higher intake of total dietary fiber (25–29 g per day) compared to a lower intake was associated with a 16% reduction in the incidence of T2D (RR = 0.84, 95% CI: 0.78–0.90), with a linear dose–response relationship (based on 17 observational studies). Furthermore, a higher intake of WGs was associated with a 33% reduction in the risk of T2D (RR = 0.67, 95% CI: 0.58–0.78) (based on eight observational studies), supporting the beneficial effects of WGs due to their high fiber content [116]. Based on the Nurses’ Health Study, the Nurses’ Health Study II, and the Health Professionals Follow-up Study, all large-scale prospective studies including a total of 194,274 participants without a diagnosis of T2D, CVD, or cancer at baseline and 18,629 subjects identified with T2D during a mean follow-up of 24 years, Hu et al. [26] explored the associations of total and individual WG intake with the risk of T2D. After dividing total grain consumption into five categories of servings a day and adjusting for lifestyle and other dietary risk factors, the pooled analysis of all three cohorts indicated that the highest intake vs. the lowest intake of total WGs was associated with a 29% lower occurrence of T2D, after adjusting for covariates (HR = 0.71, 95% CI: 0.67–0.74). For individual WG foods, comparing consumption of more than one serving per day vs. less than one serving per month, the pooled HRs for T2D were 0.81 (95% CI: 0.77–0.86) for WG cold breakfast cereal, 0.79 (95% CI: 0.75–0.83) for dark bread, and 1.08 (95% CI: 1.00–1.17) for popcorn [26]. In line with [96], total WG consumption was inversely correlated with the risk of T2D according to a nonlinear relationship showing a plateau between two and three servings a day [26]. Subgroup analysis revealed a relatively weaker inverse association between total WG intake and T2D risk in obese subjects, compared with that observed in lean and overweight participants; the chronic conditions of inflammation, dyslipidemia, and insulin resistance typical of obese individuals may counteract the benefits of WG consumption on glucose metabolism [26].

**Table 2:** Clues and pitfalls in the association between whole grain intake and risk of type 2 diabetes.

Clues	Reference	Pitfalls	Reference
WG intake (3–5 servings per day) significantly associated with a reduced risk of T2D	[96,106,111,116]	Potential overestimation due to incomplete adjustment for lifestyle and dietary factors, as well as unmeasured or residual confounding	[26,96,106,111]
Significantly inverse associations between WG bread, whole grain cereals, wheat bran, and brown rice and risk of T2D	[96,115]	Most studies conducted among Caucasian populations in the United States	[106,111]
No significant association between refined grain consumption and T2D risk	[96]	Small number of cohort studies	[96]
Whole grain intake (the highest category vs. the lowest category) significantly inversely associated with T2D occurrence	[26]	High heterogeneity in the dose–response analysis of WGs and T2D	[96]
Whole grain cold breakfast cereals and dark bread (≥1 serving per day) significantly associated with a reduced risk of T2D	[26]	No possibility to control publication bias	[96]
		Possible inadequate reporting of WG consumption from subjects	[106]
		Possible measurement errors and differences between studies in the exposure assessment	[96,111]
		Lack of a uniform definition for WG foods	[96,106]
		Wide range of whole grain intake across studies	[106]
		Possibility of false results due to the assessment of associations of WG foods simultaneously	[26]
		Findings mainly related to white health professionals	[26]
		Evidence for the association between dietary fiber and whole grain intake and the risk of T2D rated as low or moderate by the GRADE criteria assessment	[116]

Abbreviations: T2D: type 2 diabetes; WGs: whole grains.

#### 3.2.1. Whole Grain Intake and the Impact on Glycemic Control

**Figure 2:**
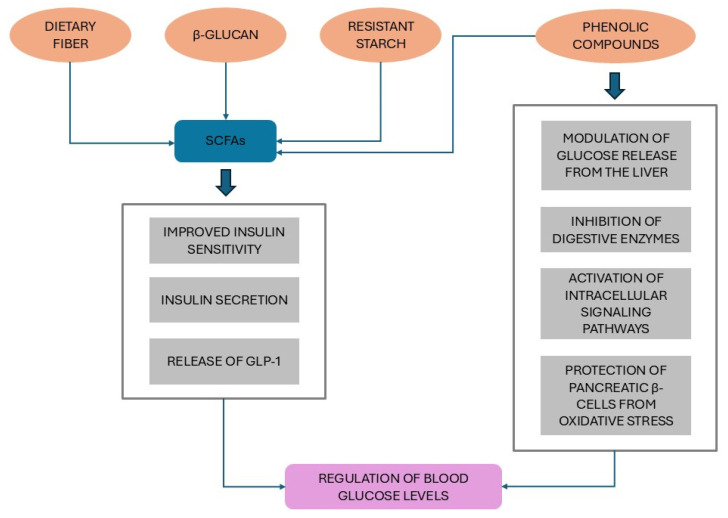
Summary of biological processes involved in glycemic control following whole grain consumption. Abbreviations: GLP-1: glucagon-like peptide-1; SCFAs: short-chain fatty acids.

The inverse association between WG intake and T2D risk can be partially explained by the regulatory effect of WG on blood glucose levels through a significant decrease in fasting glucose and postprandial glycemia, insulinemia, and HbA1c [117,118,119,120]. In particular, the significant improvement in HbA1c can account for the cumulative positive effect of WGs on postprandial glycemia [119]. A systematic review and meta-analysis of 21 RCTs showed that, in comparison to controls, WG intervention groups had significantly lower concentrations of fasting glucose, insulin, total and low-density lipoprotein (LDL) cholesterol, and lower weight gain after 4–16 weeks [111]. A meta-analysis of 14 RCTs [117] reported that the acute effects on postprandial glucose and insulin homeostasis promoted by WG intake in healthy subjects did not significantly differ from those of refined grain meals, contrary to a subsequent meta-analysis including 80 RCTs [118], which instead documented significantly lower postprandial glycemia and insulinemic response compared to refined grain foods, despite the small effect size. Furthermore, WG consumption did not significantly affect fasting insulin, a homeostatic model assessment of insulin resistance (HOMA-IR), or glucose tolerance, suggesting little or no effect on insulin sensitivity [117,118,119]. A recent meta-analysis of 25 RCTs showed that although WG intake may improve HbA1c in adults with or without risk factors for CVD, the corresponding level of evidence was moderate; therefore, the authors’ opinion was of no clear indications to recommend WG in replacement of refined grains to prevent and/or treat CVD [120]. In contrast, the meta-analysis by Ying et al. [121], based on ten prospective studies and 37 RCTs, reported that the intake of WGs was significantly associated with reduced fasting blood glucose, HbA1c, and HOMA-IR according to a dose-dependent pattern. Fiber, and in particular β-glucan, a non-starch soluble polysaccharide contained in barley, oat, and rye, increases the viscosity of the gastrointestinal tract which, by limiting the accessibility of digestive enzymes, reduces the intestinal absorption of carbohydrates, with a consequent reduction in postprandial glycemia [119,122,123]. This action on glucose metabolism probably involves the modulation of the hormone cholecystokinin, which, when secreted in the upper part of the small intestine following a meal, delays gastric emptying and enhances postprandial satiety [124]. Consistently, the intake of WG foods is significantly associated with decreased appetite and increased satiety perception compared to refined grain foods [125]. Of note, β-glucan in barley may influence the composition of the gut microbiota by increasing the abundance of specific succinate-producing bacteria and succinate, a precursor of propionate, one of the short chain fatty acids (SCFAs), which represent the main metabolites produced by the intestinal microbiota from dietary fiber and resistant starch [119,126]. Among multiple biological effects, SCFAs may improve glucose metabolism function and insulin sensitivity, thus explaining the effects of WG intake on postprandial insulinemia [119,127,128,129,130,131]. SCFAs also directly control hepatic function and increase insulin secretion, as well as promoting the release of the intestinal hormones peptide-YY and glucagon-like peptide-1 (GLP-1), two key regulators of appetite behavior, energy intake, and nutrient availability [131,132]. In particular, the incretin GLP-1 enhances the insulin response to ingested carbohydrates, thus helping to regulate blood glucose levels [133]. In addition to the high fiber content in bran and germ components, WGs contain a wide range of phytochemicals, among which phenolic acids are of great importance due to their antioxidant, anti-inflammatory, and anti-tumor properties [134]. In vitro studies have shown that phenolic acids (the most common ones in WGs include vanillic, ferulic acid, caffeic, syringic, and *p*-coumaric acids) can modulate carbohydrate and lipid metabolism and reduce insulin resistance, exerting anti-diabetic activities, i.e., stimulating insulin secretion and signaling, modulating glucose release from the liver through the control of gene expression, regulating the gut microbiota, activating intracellular signaling pathways, and protecting pancreatic β-cells from oxidative stress [119,134,135,136,137]. Furthermore, phenolic compounds (phenolic acids, anthocyanins, tannins, and flavonoids), are inhibitors of α-amylase and α-glucosidase, carbohydrate digestive enzymes that catalyze the hydrolysis of starch, maltodextrins, and other related carbohydrates into maltose and convert maltose into glucose, respectively, overall leading to strict control of postprandial blood glucose in diabetic patients and also contributing to the prevention of T2D [138] (Figure 2).

In summary, a strong body of evidence indicates a considerable decrease in the risk of T2D associated with an intake of all grains, especially wheat, accompanied by an improvement in fasting glucose concentration, postprandial glycemia, and insulin homeostasis. On the other hand, WGs do not appear to influence long-term glucose-related parameters, probably due to the study design and the types of WGs evaluated (whether a mix of WGs able to exert mutually reinforced effects or, more frequently, wheat alone). Although some studies have concluded that consuming 45 g/day of whole grains would be related to a reduction in T2D risk of at least 20% (up to 30%), not all authors agree that there is sufficient data to support the current recommendations to consume at least two portions of WGs per day for the prevention of chronic non-communicable diseases, such as T2D. Indeed, if WGs contain more fiber than refined grains, which leads to a reduced glycemic response due to the replacement of digestible carbohydrates with non-digestible fibers, the real benefits of WGs more probably lie in the ability of fibers and phenolic acids to slow down the rate of carbohydrate digestion, fibers to enhance the effects of incretin hormones, and β-glucan to modulate the composition of the intestinal microbiota. Therefore, large clinical studies with longer intervention durations and evaluations of both the type and variety of WGs and the dose-dependent relationship of individual WGs are warranted to translate acute effects on glycemia into permanent benefits in reducing the onset of T2D. A future challenge will also be understanding, in preclinical and clinical studies, which components of WGs, i.e., fiber, phenolic compounds, or bran, are mainly involved in lowering the risk of disease, while cytotoxicity tests will clarify the potential of these compounds as adjuvants or alternative medications in the treatment and management of T2D.

#### 3.2.2. Nutrigenetic Interaction Between Whole Grain and Type 2 Diabetes Genes

Among the numerous investigations evaluating gene–macronutrient interactions that may potentially predispose individuals to T2D development, one of the most widely studied is that between *TCF7L2* and intake of WGs and related dietary fiber [139]. In a systematic review and meta-analysis of 13 observational studies, the four studies examining this gene–diet interaction reported conflicting results, with a significantly increased incidence of T2D in subjects with a T allele (the risk allele) of rs7903146 and high consumption of total or cereal fiber observed in two studies and no significant associations reported in the other two investigations [139]. The diabetogenic effect of the *TCF7L2* variant could be mediated by a reduced expression and function of GLP-1, a hormone that, as also discussed in the previous section, plays a crucial role in regulating glucose metabolism [140,141,142]. Conversely, no-risk carriers of the rs7903146 CC and rs4506565 AA-genotypes exhibited a decreased risk of developing T2D in relation to high intakes of WGs and cereal fiber [142,143]. The systematic review by Dietrich et al. [20], in addition to the above findings, included a further study reporting an interaction between *TCF7L2* rs7903146 and another *TCF7L2* variant (rs12255372) and quintiles of cereal fiber intake on T2D incidence, showing that the magnitude of the association between *TCF7L2* variants and T2D incidence was greater among individuals in the higher fiber intake quintiles than among those in the lower quintiles [144]. In contrast, the case–cohort European Prospective Investigation into Cancer and Nutrition (EPIC)—InterAct study (a multi-center, prospective, cohort study of 519,978 participants from 8 European countries aimed at investigating the relationship of environmental factors, food habits and lifestyle with cancer and other chronic diseases), including 12,403 incident T2D cases and a random subcohort of 16,835 subjects, did not reveal significant effect modifications of *TCF7L2* variants in the association between cereal fiber and T2D, except for a borderline significant protective association among carriers of *TCF7L2* rs12255372 GG [145]. A higher dietary fiber intake was instead associated with a reduced incidence of T2D among carriers of the risk (T) allele in the rs10923931 variant of Neurogenic locus notch homolog protein 2 (*NOTCH2*) and among homozygotes for the risk (G) allele in Zinc-finger BED domain-containing 3 (*ZBED3*) rs445705 [144]. These SNPs are located on two genes upstream of *TCLF2* in the Wnt signaling pathway, which, as described in Section 2, plays a pivotal role in T2D development by mediating GLP-1-induced beta cell proliferation [144,146,147]. *NOTCH2* gene expression is significantly higher in diabetic patients than in healthy controls [148], while, on the other hand, NOTCH can inhibit Wnt/β-catenin activity by reducing the levels of active β-catenin in stem cells and colon cancer to regulate their proliferative state [149], although it is currently unknown how the effect of the *NOTCH* variant is related to NOTCH expression [144]. In addition to being involved in the modulation of Wnt/beta-catenin signaling, increasing levels of *ZBED3* are independently associated with insulin resistance and risk of T2D, and *ZBED3* has recently been identified as a regulator of hepatic glucose metabolism, promoting hepatic gluconeogenesis under glucagon stimulation and thus regulating T2D progression [150,151,152].

Within a prior meta-analysis of 14 cohort studies comprising around 48,000 participants of European descent, WG intake showed the strongest interaction with the glucokinase regulator gene (*GCKR*) rs780094 in relation to fasting insulin concentration [153]. Indeed, glucokinase (*GCK*) serves as a glucose sensor and maintains blood glucose homeostasis by regulating glycogen synthesis and gluconeogenesis, and *GCKR* acts as the primary regulator of *GCK*, inhibiting its enzymatic activity at low glucose levels [154]. *GCKR* SNPs (rs780093 T>C, rs780094 T>C, and rs1260326 T>C) have been related to increased glucose levels and HOMA-IR but to decreased concentrations of total cholesterol and triglycerides [155]. Conversely, the T allele of these variants has been associated with lower fasting glucose levels, reduced insulin resistance and T2D risk, and, at the same time, higher 2 h postprandial glucose and triglyceride levels [156]. Consistently, the meta-analysis by Nettleton et al. [153] found that subjects carrying one or two copies of the *C*-allele in the polymorphic locus rs780094 exhibited a weaker insulin-lowering effect (from 0.010 to 0.018 units) upon a greater WG intake than those not carrying the insulin-raising rs780094 C allele, although these relationships lost statistical significance after adjustment for covariates.

In sum, a diet rich in WGs and fibers seems to decrease fasting insulin independently of genetic variation and to have a protective effect against the onset of T2D, especially in subjects carrying the risk-free alleles of *TCF7L2.* However, it is likely that the generally inconsistent results observed are attributable to a predominance of study samples composed mainly of European ethnicities, with a concomitant underrepresentation of other ethnicities, which may influence the frequencies of variants associated with T2D and the resulting interactions with dietary factors.

### 3.3. Glycemic Index, Glycemic Load, and Risk of Type 2 Diabetes

While a substantial body of evidence supports a significant association between high-fiber whole foods and a reduced risk of T2D, with a risk ratio of 0.85 (95% CI: 0.82–0.89) observed for every 8 g more fiber consumed per day [116], low-fiber carbohydrate diets potentially have the greatest impact on postprandial circulating glucose concentration [157]. High-carbohydrate diets result in a high glycemic index (GI—a classification of carbohydrate foods based on their ability to increase blood glucose concentration in comparison with reference food and, consequently, insulin requirements) and glycemic load (GL—a global score that indicates the glycemic response induced not only by the GI but also by the total amount of carbohydrates ingested and influenced by insulin resistance and related factors such as genetics, overall diet, lifestyle, and physical activity), which may plausibly contribute to the positive association between carbohydrate intake and the risk of T2D [27,158,159]. In both in vivo and short-term epidemiological studies, the intake of high-GI carbohydrates causes, on the one hand, pancreatic exhaustion due to increasing insulin demand and glucose intolerance and, on the other hand, an increase in free fatty acid release in the late post-prandial state, which may lead to insulin resistance [159]. Data from large prospective cohort epidemiological studies further suggest that a high-GL diet carries a significantly higher risk of T2D than a low-GL diet [159,160]. Additionally, a number of meta-analyses have explored the relationship between dietary GI and GL in relation to T2D risk, albeit with somewhat conflicting results and substantial limitations (Table 3). According to a systematic review and meta-analysis of 13 prospective cohort studies published between 1997 and 2010 including 530,875 participants, the pooled adjusted RR of T2D comparing the highest to the lowest category of GI was 1.16 (95% CI: 1.06–1.26), while the comparison between the highest and the lowest GL exposure was associated with an overall 20% increased risk (RR = 1.20, 95% CI: 1.11–1.30) [161]. Importantly, the two relationships remained significant even in the sensitivity analyses, and no publication bias was observed [161]. A meta-analysis of 22 cohort studies published in the years 1992–2011, for a total of 1,171,865 subjects in the cohorts [27], reported a significantly positive association between the intake of total dietary carbohydrates and incident T2D, with an increment of risk by 11% (RR = 1.11, 95% CI: 1.01–1.22, *p* = 0.035). However, the statistical significance was not observed in subgroup analyses evaluating individual sugars (i.e., fructose, glucose, lactose, maltose, sucrose) [27]. In addition, the effect was attenuated when considering only female participants or male and female participants [27]. A dose–response meta-analysis including a total of 21 prospective cohort studies from 24 publications (years 1990–2012), involving a total of 542,495 subjects, confirmed the previous findings, showing an 8% increased risk of T2D (RR = 1.08, 95% CI: 1.02–1.15, *p* = 0.01) for each 5-unit increase in GI (data extracted from 15 publications) [162]. The association between GL and T2D risk was slightly weaker, with a pooled RR estimate of 1.03 (95% CI: 1.00–1.05, *p* = 0.02) for every 20-unit increase in GL (data retrieved from 16 publications) [162]. In contrast, no excess risk of T2D was observed per 50 g of total daily carbohydrate intake (RR = 0.97, 95% CI: 0.90–1.06), although a substantial heterogeneity was detected between studies (*n* = 8) [162]. This unexpected finding may reflect differences in the amount, main sources, and types of carbohydrate consumed among different cohorts of subjects, including different proportions of men and women and diverse lifestyles (e.g., more active subjects may have a higher daily carbohydrate intake) [162]. Additionally, the exposure assessment was based on individual responses to the food frequency questionnaire (FFQ), which is not specifically designed for GI and GL, and the assignment of GI values to food items in FFQ may have potentially led to measurement errors [162]. In addition, variables such as cooking methods, storage duration, and combination of food items within a meal (e.g., co-ingestion of fat and protein) may affect the GI of an entire meal [162,163]. Sluijs et al. [163], who investigated the association between GI, GL, and digestible carbohydrates (all divided into quartiles) and T2D risk within a case–cohort study nested in the large-scale, prospective, multi-center InterAct EPIC study, including 12,403 incident T2D cases and a random subcohort of 16,235 subjects, did not find any statistically significant association in adjusted analyses, suggesting that the impact of GI and GL may have been overestimated in the initial studies [163]. The systematic review and meta-analysis by Livesey et al. [164] (24 prospective studies for a total of 7.5-million-person years of follow-up), showed that GL represents a key determinant in contributing to the incidence of T2D. Indeed, the authors reported a 45% increased risk (RR = 1.45, 95% CI: 1.31–1.61, *p* < 0.001) for a 100 g rise in GL (corresponding to the consumption of 250 g carbohydrate, the usual intake in Western diets, with a GI of 40) after adjustment for covariates [163]. In particular, the dose–response relationship between GL and T2D was stronger in females, in subjects of European American ethnicity, and when the dietary instrument had greater validity, whereas the duration of follow-up, which is related to the duration of exposure to GL and to the risk of developing diabetes, did not appear to influence T2D risk [164]. An updated meta-analysis of prospective studies published up to 2013 (*n* = 14) and including 15,027 cases of incident T2D during 3,800,618 person-years of follow-up, estimated an increased risk of T2D by 19% (RR = 1.19, 95% CI: 1.14–1.24) and 13% (RR = 1.13, 95% CI: 1.08–1.17) associated with the highest categories compared to the lowest categories of GI and GL, respectively [165]. In a subsequent systematic review and meta-analysis on the relationship between GI, GL, and T2D including only studies that applied valid dietary instruments (i.e., energy-adjusted deattenuated correlation coefficients for carbohydrates > 0.55 when assessing the risk of coronary heart disease in relation to GI and GL), Livesey et al. reported a RR of 1.27 (95% CI: 1.15–1.40, *p* < 0.001) for the T2D–GI risk association (*n* = 10 studies) and a RR of 1.26 (95% CI: 1.15–1.37, *p* < 0.001) for the T2D-GL risk relationship (*n* = 15 studies) [166]. The authors hypothesized the possibility of an underestimation of these effects since a subset of studies that clinically ascertained T2D and not through self-reporting yielded higher RR estimates [166]. Furthermore, an increased risk of 87% (RR = 1.87, 95% CI: 1.56–2.25, *p* < 0.01) and 89% (RR = 1.87, 95% CI: 1.66–2.16, *p* < 0.01) was observed for the T2D–GI association between 47.6 and 76.1 GI units and for the T2D–GL association between 73 and 257 g/d GL in a 2000 kcal diet, respectively [166]. No significant differences were reported between female and male participants in the GL-T2D relationship, while, in accordance with [164], subjects of European ancestry were significantly associated with both GI and GL and met both criteria of interest for public health (RR > 1.20 and lower 95% confidence limit > 1.10) [166]. Additionally, the results of this meta-analysis met all nine Bradford Hill criteria, a group of guidelines used to verify causality between a risk factor and an outcome, indicating that GI and GL have a causal role in the incidence of T2D [166,167]. An updated meta-analysis including ten studies (publication years 2015–2021), three of which were performed in Asia, failed to find any significant association between carbohydrate intake and the risk of T2D (overall RR = 1.07, 95% CI: 0.94–1.21), consistent with the findings of [162], although a significant association was detected when considering only Asia-based studies (RR = 1.29, 95% CI: 1.15–1.45) [168]. However, although Asian populations are characterized by high carbohydrate intake and reduced insulin-releasing capacity, the difference in follow-up duration between Asian and non-Asian samples could have influenced this result [168]. A recent prospective cohort study, with a median follow-up of 13.6 years and including 161,872 participants, reported a significantly positive association between the intake of starch and the risk of T2D (HR = 1.31, 95% CI: 1.16–1.48, *p* < 0.0001 by comparing the fifth with the lowest quintile) [169]. Considering various food sources, a higher intake of carbohydrates from starchy vegetables was associated with a 19% increased risk of T2D (fifth vs. first quintile, HR = 1.19, 95% CI: 1.09–1.31, *p* = 0.0003), corroborating the importance of reducing the consumption of refined grains and starchy vegetables [169].

The overall results indicate that diets characterized by high GI and GL are generally strongly associated with the incidence of T2D in healthy subjects, with RRs reported to be between 1.08 and 1.27 and between 1.03 and 1.26 in the T2D-GI and T2D-GL relationships, respectively, which, despite the small excesses, may translate into important implications for public health. Conversely, the association between carbohydrate intake and T2D risk appears somewhat controversial. However, in this framework, the assessment of this relationship should not only analyze the daily amount and type of carbohydrates, but also the circadian timing of food intake, which could be related to a greater risk of cardiometabolic diseases. Furthermore, the generally high heterogeneity between studies reflects a wide range of exposures reported across publications due to the variety of dietary assessment tools (there are more than 150 different databases worldwide), which makes it difficult to compare data collected in different countries, leading to misclassification of dietary intake and, consequently, to a possible overestimation of the association between dietary intake and T2D risk. Therefore, future studies should use standardized methods for exposure assessment to minimize systematic errors. At the same time, studies conducted in certain geographic areas, such as Africa and South America, are not yet sufficiently explored, while research involving susceptible subject groups, like individuals with high BMI and/or a family history of diabetes or cardiometabolic diseases, is warranted.

#### 3.3.1. Carbohydrate Intake and the Impact on Glycemic Control

**Figure 3:**
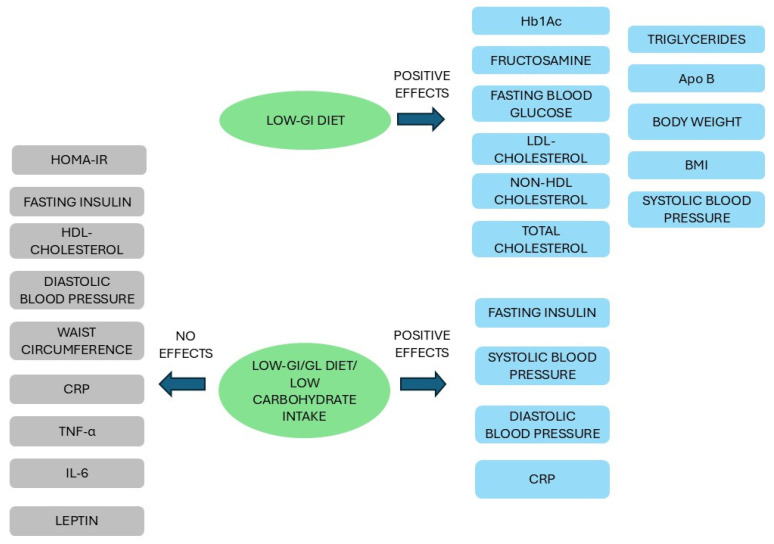
Effects of carbohydrate intake on blood parameters and other key biomarkers associated with glycemic status. Abbreviations: apo B: apolipoprotein B; BMI: body mass index; CRP: *C*-reactive protein; GI: glycemic index; GL: glycemic load; Hb1Ac: glycated hemoglobin; HDL: high-density lipoprotein; HOMA-IR: Homeostatic Model Assessment for Insulin Resistance; IL-6: interleukin 6; LDL: low-density lipoprotein; TNF-α: tumor necrosis factor alpha.

As discussed in the previous section, while GI and GL play a substantial role in contributing to the incidence of T2D, a lower GI and a higher intake of dietary or cereal fiber result in an additive decrease in T2D risk [167]. In fact, a series of meta-analyses of RCTs have underscored the beneficial effects of a low-GI diet on glycemic control in diabetic patients, as compared with high-GI diets, with significant differences in HbA1c and fructosamine (also known as glycated serum albumin, a marker of glycemic control up to six weeks) [116,170,171,172,173,174], fasting blood glucose [172,173,174], and other established cardiometabolic risk factors including LDL cholesterol, non-high-density lipoprotein (HDL) cholesterol, total cholesterol, apolipoprotein B, triglycerides, body weight, BMI, and systolic blood pressure [116,172,173,175,176]. Low-GI/GL diets or low carbohydrate intake did not affect either HOMA-IR, HDL cholesterol, waist circumference, diastolic pressure [172,173,176], nor inflammatory biomarkers such as *C*-reactive protein (CRP), tumor necrosis factor alpha, interleukin-6, or leptin, which can be involved in the development of insulin resistance and subsequently lead to T2D [177]. Conversely, a low-GI/GL diet is inversely associated with fasting insulin, both systolic and diastolic pressure, and CRP [178,179] (Figure 3). Overall, these findings, which confirm the effectiveness of the low-GI/GL dietary pattern strategy in producing a relevant improvement in the major targets of glycemic control in people with prediabetes or diabetes and with a concomitant reduction in the number of hypoglycemic episodes, are in line with the recommendations of the American Diabetes Association, which encourage the consumption of non-starchy vegetables, whole fruits, legumes, and WGs [173,180]. Furthermore, based on a recent meta-analysis including 23 published and unpublished RCTs for a total of 1357 patients with T2D, moderate to little evidence suggests that subjects adhering to a low-carbohydrate diet (LCD) for six months may achieve higher diabetes remission rates (around 30%) concurrently with reduced medication use, increased weight loss, and improved triglyceride profile when compared to patients undergoing low-fat diets, which are among the most common diets recommended for the management of T2D [181]. These beneficial effects tend to decline after 12 months, when increased levels of LDL were observed, while long-term LCD can lead to adverse outcomes, including a 20% increase in mortality, suggesting that LCD represents an effective strategy when used only for short-term periods [181]. It should be noted, however, that these results are profoundly influenced by the source of the macronutrient replacement, as excess of mortality can be detected when carbohydrates were exchanged for animal-derived fat or protein [98,181]. A recent 16-year prospective cohort study revealed that replacing 5% energy from refined grains or starchy vegetables with an equal amount of WGs or non-starchy vegetables was associated with a significant decrease in the risk of T2D, ranging from 8 to 17%, highlighting the importance of food sources in the primary prevention of T2D [169].

#### 3.3.2. Nutrigenetic Interaction Between Carbohydrates and Type 2 Diabetes Genes

The variant rs2943641 of *IRS1*, which plays a central role in the insulin signaling pathway, appears to interact with macronutrient intake in modulating T2D risk [182]. rs2943641 *IRS1* interacts with sex and carbohydrate intake, and females carrying the T allele exhibit a reduced risk of developing T2D in the lowest tertile of carbohydrate intake [183]. A 2-year RCT comparing the effects of energy-restricted diets on body weight in overweight and obese subjects reported a significant interaction between *IRS1* rs2943641 genotype and dietary groups on changes in weight, insulin resistance, and HOMA-IR after adjustment for covariates [184]. In fact, at 6 months, participants in the highest-carbohydrate-intake group and with the CC genotype showed a greater decrease in insulin levels than subjects who did not have this genotype, while an opposite effect was observed in the group with the lowest carbohydrate diet [184]. A similar interaction between the *IRS1* rs2943641 genotype and the carbohydrate diet groups was also found for changes in HOMA-IR [184]. At 2 years of follow-up, the effect of the CC genotype on changes in insulin and HOMA-IR remained significant in the highest-carbohydrate-diet group, demonstrating that the *IRS1* variant rs2943641 may improve insulin resistance in response to weight-loss diets [184]. In the study by Zheng et al. [185], two *IRS1* variants (rs7578326 and rs2943641) were tested for their associations with insulin resistance, T2D, and MetS, as well as their interactions with diet in two populations of different ancestries: the first one (Genetics of Lipid Lowering Drugs and Diet Network—GOLDN) composed of 820 subjects, all of European descent, and the second one (Boston Puerto Rican Health Study—BPRHS) composed of 844 participants mostly of European descent (57.4%) and for the remaining parts African (27.4%) and Native American (15.4%). Meta-analysis revealed a lower risk of impaired fasting glucose, T2D, and MetS among T-allele homozygotes compared to *C*-allele carriers in the SNP rs2943641 and of T2D and MetS in T-allele homozygotes compared to *G*-allele carriers in the rs7578326 variant [185]. Both variants interacted with dietary carbohydrates in modulating the risk of HOMA-IR. In the GOLDN population, SNP rs7578326 G-allele carriers and rs2943641 T-allele carriers had significantly lower HOMA-IR than noncarriers when the short fatty acid (SFA)-to-carbohydrate ratio was low, supporting a protective effect of a high-carbohydrate and low-fat diet against T2D, as previously observed in [184], but in contrast with findings from [183], probably due to differences in study design and dietary intake ranges [183]. Similarly, in the BPRHS population, rs7578326 G-allele carriers showed lower HOMA-IR than A-allele homozygotes only in the case of a low content of dietary monounsaturated fatty acid and glycemic load [185]. Furthermore, while in the BPRHS population no significant interactions between *IRS1* variants and carbohydrates influenced the risk of T2D, impaired fasting glucose levels and MetS in the GOLDN population subjects with the rs7578326 G allele and those with the rs2943641 T-allele had a reduced risk of MetS compared to AA and CC carriers, respectively, only when SFA-to-carbohydrate ratio was ≤0.24, thus suggesting the importance of developing specific dietary recommendations in different populations [185]. Consistently, Gao et al. [109], using detailed dietary data from 120,343 participants from the UK Biobank study, in which 2878 participants developed T2D over 8.4 years of follow-up from the latest dietary assessment, did not identify any significant association between a dietary pattern characterized by a high intake of sugar-sweetened beverages, fruit juice, table sugars and preserves, in the context of a low intake of high-fat cheese and butter, and incidence of T2D. Low SFA levels and concomitant consumption of adequate amounts of fiber from fruits and vegetables may account for most of this effect, although other unconsidered nutrients may explain the remaining variability implicated in the disease pathway [109].

To sum up, specific haplotypes of *IRS1* variants are associated with a reduced risk of insulin resistance and T2D, and this relationship appears to be modulated by carbohydrate intake and the SFA-to-carbohydrate ratio, in line with recent data from a large prospective observational study. Nonetheless, these findings should be confirmed by studies performed across different populations to ensure reproducibility and increase statistical power, with repeated exposure measurements that also include nutritional biomarkers and using experimental settings that allow for dietary manipulation in subjects with different genetic profiles, in order to definitely establish a causal association between gene–diet interactions and the incidence of T2D.

## 4. The Carbohydrate–Epigenetics Relationship in Type 2 Diabetes

Epigenetics, which refers to the inheritable and reversible changes in gene expression without alterations in DNA sequences, represents an interface between endogenous (pathological conditions) and exogenous environmental determinants (diet, lifestyle, toxins) and the genome, which is able to affect cells, tissues, or a whole organism and can transmitted to the next generation [24,186,187]. Epigenetic phenomena, which are implicated in a wide range of cellular processes such as cell differentiation, parental imprinting, genomic stability, and X-chromosome inactivation, include changes in DNA methylation, covalent modifications of histones—both of which can modify chromatin structure and thus modulate access to transcription factors—and non-coding RNA interference, which controls gene expression at the RNA level [187,188,189]. In DNA methylation, the most important epigenetic mark, a methyl group, is mainly added in the 5′ position of the cytosine residues of cytosine–guanine dinucleotides (CpG), frequently forming dense repeat sequences, known as CpG islands, in the promoter region [24]. DNA methyltransferases (DNMT1, which maintains methylation during DNA replication and de novo enzymes, DNMT3a, and DNMT3b) are responsible for the transfer of methyl groups to DNA, which generally results in the inhibition of gene expression when methylation occurs in regions close to the transcription start site and in the enhancer region [24,186,187]. Conversely, the hydroxymethylation of 5-methyl-cytosine, catalyzed by DNA demethylases termed ten-eleven translocation proteins, leads to the activation of transcriptional activity [24,187]. If DNA methylation is a reversible modification with a crucial role in various physiological processes, aberrant DNA methylation profiles caused by genetic mutations or environmental factors may contribute to the occurrence of diseases including autoimmune diseases, cancer, and metabolic and neurological disorders [24,188]. Modifications of histones (globular proteins that serve to package and organize DNA within the nucleus [190]) are another form of epigenetic information that typically occurs post translationally at their *N*- and *C*-terminal tails [191]. They comprise methylation, acetylation, ADP-ribosylation, glycosylation, phosphorylation, SUMOylation, and ubiquitination, which, changing the electronic charge and structures of these histone tails, may cause alterations in chromatin status, resulting in the activation or silencing of expression [192,193,194]. Accumulating data indicate that histone modifications are closely associated with the development of inflammatory diseases such as T2D, Alzheimer’s disease, asthma, atherosclerosis, inflammatory bowel disease, and psoriasis [194]. Non-coding RNAs, i.e., RNAs that are not translated into proteins, have a regulatory role and can be divided into two categories based on size: short chain non-coding RNAs (micro-RNAs—miRNAs, piwi-interacting RNAs, and small interfering RNAs—siRNAs) and long non-coding RNAs (lncRNAs) [195]. They can act at transcriptional levels alone, leading to gene silencing (miRNAs and siRNAs), or both at transcriptional and post-transcriptional levels by interacting with enhancers, promoters, chromatin-modifying complexes, and miRNAs (lncRNAs) [195,196]. By modulating gene expression, miRNAs have the ability to govern various cellular processes and, consequently, their dysregulation has been associated with diseases such as autoimmune and inflammatory diseases, cardiovascular disease, and neurodegenerative disorders [197]. LncRNAs have also progressively emerged as regulators of the inflammatory response through the precise control of inflammation-related gene expression and are therefore hypothesized to play a crucial role in diabetic retinopathy, a serious complication of T2D, whose hallmarks include inflammation and apoptosis [198].

### 4.1. Epigenetics in Type 2 Diabetes: The Role of DNA Methylation

As reported in the previous sections, genetic susceptibility in T2D does not cover the total amount of cases, and genetic variants account for approximately 20% of heritability. Therefore, environmental and lifestyle factors, contributing to epigenetic changes, may explain the so-called missing heritability in T2D and differences in disease susceptibility between individuals [24,199,200]. A growing body of evidence indicates that the dysregulation of expression of non-coding RNA, particularly those involved in insulin secretion and glucose and lipid metabolism, can contribute to T2D development [201,202], while histone modifications appear to be involved in the pathophysiology of T2D, affecting the development of pancreatic β cells and insulin release, and complications of T2D [203,204]. However, CpG methylation has been the most widely studied epigenetic phenomenon to date, with dozens of publications showing the association between changes in DNA methylation profile and risk of T2D. Initial studies evaluating DNA methylation in candidate genes for T2D (e.g., *IRS*, *GLP1-R*, encoding the receptor for GLP-1, *PPARGC1A*, encoding the peroxisome proliferator-activated receptor γ coactivator-1 alpha, a transcriptional co-activator involved in cellular energy metabolism [205]) found that the DNA methylation level in pancreatic islets of T2D subjects was lower than that of non-diabetic control, with a consequent reduced expression of these pivotal genes, which can explain the impaired insulin secretion, high glucose, and Hb1Ac levels [189]. The introduction of the Illumina sequencing array, which presents cost-effectiveness and overall good accuracy, has allowed for identification of methylation sites in thousands of genes expressed in other tissues, such as the liver, skeletal muscle, and adipose tissue [206,207]. Beyond islet cells, increased methylation and downregulation of *PPARGC1A* have been detected in the skeletal muscle and adipose tissue of insulin-resistant and obese individuals at high risk of T2D [208,209]. A recent study documented substantial changes in the methylation patterns of 921 genes expressed in skeletal muscles and involved in calcium/lipid metabolism and mitochondrial function in obese individuals 52 weeks after bariatric surgery, with a concomitant improvement in insulin sensitivity [210]. Overall, while these data suggest that DNA methylation processes are related to the reprogramming of gene expression in response to metabolic changes, it is still unclear whether these alterations directly influence insulin action or whether the improvement in insulin sensitivity is attributable to weight loss alone [210,211]. Within a systematic review including 47 studies for a total of 10,823 participants and 3358 T2D cases, Muka et al. [207] reported no consistent association between global DNA methylation and T2D and glycemic traits, although the cross-sectional design of most studies, together with the small sample size, the frequent lack of adequate adjustment for confounders, and the lack of standardized approaches in epigenome-wide association studies (EWASs), may have considerably affected the results. A subsequent systematic review [212] selected 37 studies that overall highlighted reproducible differential methylation in a set of genes in blood involved in glucose and lipid metabolism and energy intake and expenditure (e.g., *FTO* encoding a 2-oxoglutarate-dependent nucleic acid demethylase whose variation in expression is associated with the regulation of food intake and energy balance [213], *TCF7L2*) as well as in insulin secretion and function (e.g., *SLC30A8*, which encodes a zinc transporter expressed primarily in pancreatic β-cells and which plays a pivotal role in maintaining glucose homeostasis [214], and *GIPR*) in different population groups. Of note, considering that up to 25% of all SNPs in the genome contain CpG sites undergoing methylation or demethylation, a study included in this review suggests that SNPs associated with 20 genes may also cause their differential methylation, thus contributing to the pathogenesis of T2D [212]. A systematic review of 19 EWASs (of which 18 out of 19 had a cross-sectional design) assessing the association between DNA methylation and T2D or glycemic traits identified differentially methylated sites in the blood: *TXNIP*, which encodes thioredoxin-interacting protein, representing the main regulator of glucose balance [215]; *ABCG1*, encoding a member of the ATP-binding cassette protein family implicated in cholesterol transport and glucose homeostasis [216]; *CPT1A*, which encodes for the enzyme carnitine palmitoyltransferase 1, which initiates the mitochondrial oxidation of long-chain lipids [217]; and *SREBF1*, which encodes sterol regulatory element-binding proteins, transcription factors involved in the regulation of lipid and glucose metabolism [218]. These CpGs were associated with T2D status, sustained hyperglycemia levels, fasting blood glucose, and insulin resistance independently of ethnicity and environmental exposures, although the cross-sectional design of most studies precludes determining whether changes in DNA methylation precede the onset of T2D [199,219]. Furthermore, some included studies reported significant methylation differences at liver and pancreas loci in individuals with T2D compared to control individuals butshowed no overlap with blood-based EWAS results, thus indicating tissue-specific changes [199]. Within an incident T2D case–cohort study nested within the population-based EPIC-Norfolk study, a prospective cohort study recruiting 25,639 individuals, Cardona et al. [220] confirmed the results of [199], identifying 18 methylation variable positions in whole blood strongly associated with incident T2D, of which the most robust involved *TXNIP* (decreased methylation), *ABCG1*, and *SREBF1* (both increased methylation). Additionally, the authors reported a causal role of methylation at the cg00574958 site in *CPT1A* in T2D [220]. Therefore, these DNA methylation markers are plausibly related to T2D development via glucose- and obesity-related pathways that exert their effects many years before the disease onset [220]. A meta-analysis of EWAS results from five European cohorts, with a total 1250 cases and 1950 controls and based on blood samples collected 7–10 years before the diagnosis of T2D, identified 76 CpGs that were significantly and differently methylated in subjects with incident T2D compared to healthy subjects [220]. Nonetheless, the adjustment for BMI alone, or for BMI, smoking, and years of follow-up, reduced the significant associations to only 4 and 3 genes, respectively (including *TXNIP* and *ABCG1*), suggesting that BMI may influence the relationship between DNA methylation and T2D incidence [221]. A recent systematic review including 32 studies found that overall, among a total of 130 selected differentially methylated genes across tissues (i.e., adipose tissue, blood cells, liver, pancreatic isles) between T2D cases and healthy controls, *ABCG1* and *TXNIP* (hypermethylated in blood), *PPARGC1A* (hypermethylated in skeletal muscle), and *PTPRN2* (hypermethylated in blood, hypomethylated in adipose tissue, and encoding the protein tyrosine phosphatase receptor type N2 that is involved in insulin response to glucose levels [222]) showed a differential methylation pattern in more than one study [223].

Based on current data, epigenetics could explain the apparent missing heritability of T2D, with epigenetic alterations in genes implicated in insulin secretion (*GIPR*, *SLC30A8*, *PTPRN2*), glucose homeostasis and lipid metabolism (*TXNIP*, *CPT1A*, *SREBF1*, *ABCG1*), and energy balance (*FTO*, *PPARGC1A*) in blood, as well as in insulin-responsive tissues. These epigenetic patterns, by modifying the expression of crucial genes, some of which are candidate genes for T2D, potentially contribute to T2D pathogenesis, as documented in recent prospective studies, pointing to potential development of novel strategies of primary prevention and possible treatment of this disease. However, the large variability between studies due to the different approaches used to measure DNA methylation, the frequently applied cross-sectional or case–control design, and the lack of key confounding covariates such as BMI, alcohol consumption, lifestyle, and presence of comorbidities in the epigenetic analysis prevent direct comparison of the results and inference of a causal relationship, making it necessary to perform longitudinal studies employing standardized analysis methods and repeated measurements of methylation.

### 4.2. Carbohydrate–Epigenetics Interactions in Type 2 Diabetes

In Section 3.2.2 and Section 3.3.2, we discussed how the interactions between carbohydrate intake and certain gene variants related to T2D development may modulate this relationship. Likewise, different diet regimens, such as high-fat feeding and global caloric restriction applied in experimental and human interventional studies, have been widely established to impact on the human epigenome (reviewed in [24,189]). An excessive accumulation of fat induces a myriad of metabolic abnormalities, including insulin resistance, dyslipidemia, β-cell dysfunction, prediabetes, and T2D [224]. Therefore, obesity, especially when it involves increased abdominal and intra-abdominal fat distribution, represents a major contributing factor to the increasing worldwide prevalence of T2D [224]. Maternal diet (high-fat, low-protein, or nutrient-restriction patterns) can cause great epigenetic effects in both mothers and, given the high susceptibility of developmental stages like the intrauterine period, in offspring, increasing the possibility of developing metabolic disorders including obesity and T2D in adulthood [186]. Maternal caloric restriction, including vitamin B_12_ and folate deficiency, may alter the methylation profile in the promoter region of hepatic genes, resulting in increased adiposity and insulin resistance in the offspring in later life [225]. The methyl groups for DNA methylation come from S-adenosylmethionine, the second most common enzymatic cofactor after ATP, which in turn originates from the essential amino acid methionine via hepatic one-carbon metabolism [226,227]. Indeed, vitamin B_12_ acts as a cofactor in the methionine synthase reaction, which catalyzes the conversion of homocysteine to methionine and is dependent on methylfolate, which provides methyl groups for the synthesis of S-adenosyl methionine [225]. Consistently, diabetic subjects with hepatic hypomethylation are characterized by reduced circulating folate levels compared to non-diabetic subjects, while, conversely, folate intake in young adulthood is significantly inversely associated with the incidence of T2D, along with plasma homocysteine and insulin [228,229]. Therefore, any dietary disturbance, which may also involve fluctuations in methionine concentration, can have an impact on DNA methylation and, if occurring during intrauterine life, leaves a signature and manifests its effects during subsequent generations when the altered gene expression may contribute to the pathogenesis of metabolic disorders such as T2D [226].

*CPT1A* is among the major genes subject to differential methylation in T2D, with a possible causal role in this condition. Extensively expressed in the liver, in addition to the adipose tissue, fibroblast, kidney, lymphocytes and pancreas, *CPT1A* catalyzes the conversion of long-chain acyl-coenzyme (Co)A to acyl-carnitine that, once entering the mitochondrial matrix, is converted to acyl-CoA, which in turn participates in the acid β-oxidation cycle [230,231]. Therefore, differential expression of *CPT1A* may explain its crucial role in a series of physiological processes, including glucose synthesis, insulin release, and appetite control [232]. A genome-scale analysis conducted on sixty subjects and paired-sex sibling controls demonstrated that exposure to famine during early gestation (in the setting of Dutch Hunger Winter at the end of the World War II) was associated with differential DNA methylation at open chromatin regions and enhancers in six genes, including *CPT1A* [233]. Although the authors could not rule out that changes in DNA methylation may have occurred over the six decades since the exposure and DNA measurements, they found no influence of age and lifestyle on DNA methylation patterns [233]. In early human studies, while high levels of methylation at *CPT1A* cg00574958 have been associated with a decrease in BMI and waist circumference [234] as well as in fasting triglycerides, high and medium levels of very low-density-lipoprotein cholesterol and in the small subfraction of LDL only [235,236], a lower methylation at *CPT1A* was observed following a high-fat meal [237]. Within an EWAS including 846 participants of European descent, Das et al. [238] also observed a significantly inverse relationship between methylation at cg00574958 and cg17058475 and the presence of MetS, and these results were also replicated in younger subjects of both European American and African American ancestry. Since reduced *CPT1A* methylation and the resulting increase in gene expression appear to be related to improved transport and metabolism of triglycerides and a more favorable lipid profile, it is plausible that MeS is associated with hypermethylation at this site [235,238]. Therefore, the unexpected result of Das’ study could be attributed to a change in *CPT1A* methylation promoted by MetS itself [238]. On the other hand, *CPT1A* is regulated by various environmental factors, including dietary conditions [230]. Indeed, the combination of a high-fat diet during early life and adulthood in rodents induces increased *CPT1A* expression and hepatic lipid accumulation [239], as previously observed by Lai et al. in an EWAS [237]. Conversely, experiments in rodents reported a significant relationship between high fructose intake and increased DNA methylation at *CPT1A* promoter regions [240]. Interestingly, *CPT1A* is a target of PPARα, whose transcriptional levels are also thought to be reduced due to promoter hypermethylation following fructose consumption, and this can also explain the resulting decreased levels of CPT1A [235,240]. Considering that PPARα is a pivotal transcription factor functioning as a lipid sensor in the liver and thus regulating the expression of numerous genes involved in metabolic processes, including peroxisomal and mitochondrial β-oxidation, epigenetic variations within *PPARα* lead to a downregulation of β-oxidation activity, promoting the development of pathological conditions such as hyperlipidemia, obesity, and T2D [240,241]. Previously, Nagai et al. [242] showed that high-fructose feeding suppressed hepatic *PPARα* expression in rats, also observing a direct effect of fructose on *PPARα* expression in primary cultured hepatocytes, which causes cellular lipid accumulation. Conversely, fenofibrate, an activator of *PPARα* and a triglyceride-lowering drug, increases the PPARα protein content, also affecting the expression of its target genes, such as those implicated in β-oxidation [242]. A recent study by Lai et al. [232] evaluated the association between both carbohydrate and fat consumption and cg00574958 methylation at *CPT1A* and the risk of metabolic disease in three populations, for a total of nearly 4000 subjects. In all the populations analyzed and even with a stronger effect, the meta-analysis of the three populations revealed that carbohydrate intake was significantly and positively correlated with *CPT1A*-cg00574958 methylation, resulting in decreased gene expression [238]. In contrast, fat intake was negatively associated with the level of gene methylation, as also observed in [238]. In particular, the strongest association was observed between *CPT1A*-cg00574958 methylation and total carbohydrate intake, followed by consumption of complex and simple carbohydrates [238]. *CPT1A*-cg00574958 expression was also significantly positively associated with fasting glucose and triglyceride levels and BMI, indicating that the methylation of *CPT1A* is a mediator of the effects of carbohydrate intake on metabolic parameters [238]. On the other hand, carbohydrate intake had a significant yet negative effect on all the metabolic traits assessed, i.e., glucose, triglycerides, BMI, hypertension, MetS, and T2D, due to the mediating action of *CPT1A* methylation [238].

Therefore, several lines of evidence suggest that not only does *CPT1A* have a possible causal role in T2D, an effect mediated by differential methylation patterns, but carbohydrate intake is crucial in inducing *CPT1A* methylation, thereby influencing the risk of developing MetS and T2D. These findings are supported by both experimental and observational studies, although the absence of clinical controlled studies prevents ascertaining a causal relationship between high-carbohydrate diets and *CPT1A* methylation.

## 5. Conclusions and Future Perspectives

**Figure 4:**
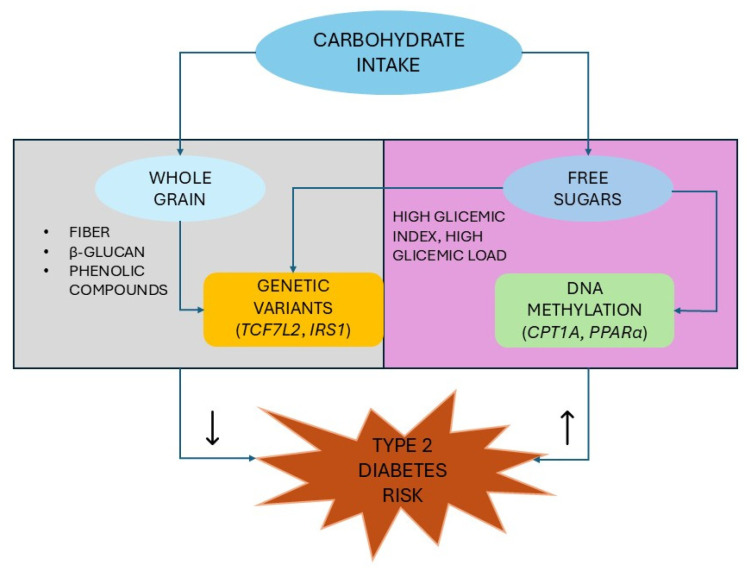
Summary of the interconnections between carbohydrate intake and genetics and epigenetics in the onset of type 2 diabetes.

The current state of knowledge identifies nutrigenetics, nutrigenomics, and nutritional epigenetics as key components in the modulation of the risk of a complex multifactorial condition such as T2D, in which genetics and environmental factor interactions play a key role in disease development. In recent decades, research attention has increasingly turned to diet and, in general, lifestyle as critical determinants in contributing to the risk of T2D. Carbohydrates, which in healthy dietary patterns such as the Mediterranean diet represent the main macronutrient, have been differently associated with diseases and mortality risk depending on both the amount and the type of carbohydrates ingested. While WGs, containing valuable elements including fiber, β-glucan, and polyphenols, which lower carbohydrate digestion by inducing the action of incretin hormones and, at the same time, promote insulin secretion and modulate the composition of the intestinal microbiota, have generally been linked to a reduced incidence of T2D, the opposite effect is observed following meals characterized by elevated GI and GL, despite conflicting results due to heterogeneity between studies (Figure 4).

From this foundation, a significant branch of scientific research has started focusing on so-called “functional foods” [243], foods that provide a scientifically proven specific health benefit beyond their nutritional format [244]. Such compounds allow for enhancing the health and well-being status of an individual, both in case they are not affected by clinically relevant disorders or if they present diseases like T2D. In that, functional foods can deliver a significant benefit to the health status of patients with T2D, but to fully enter the diet of such individuals they must face cultural hurdles and, in many cases, issues related to their palatability [245]. In such a framework, sensory and emotional analysis should be totally included in the overall pipeline of functional food production and functionalization, with traditional methods of sensory analysis placed side-by-side with instrumental measurements of chemicals contained within food and by emotional analysis, having the potential to reveal the implicit psychophysiological activation brought about by the compound and the related chemosensory stimuli on the end-users [246,247].

Therefore, if a WG-based diet is crucial in controlling glycemic response and, in general, in preventing the onset of non-communicable chronic diseases, future intervention studies should be performed both to improve the understanding of which component in WGs is mainly involved in reducing T2D risk and to better clarify which genes variants are involved in this process. *TCF7L2* represents the main gene implicated in the development of T2D, with the rs7903146 variant associated with one in five cases and, in the presence of TT genotype, appearing to interact with a high consumption of cereal fiber. Likewise, one or two copies of the C allele in *GCKR* rs780094 confer an increased risk of developing T2D in the case of WG consumption. In contrast, other *TCF7L2* SNPs, together with selected variants of *NOTCH2* and *ZEBD2*, are associated with a decreased risk of T2D in relation to a higher WG or dietary fiber intake, although with limited evidence, suggesting that fiber and other micronutrients contained in WGs have a protective effect against the incidence of T2D independently of genetic variations. On the other hand, a relevant interaction has been reported between the *IRS1* rs2943641 CC variant and high carbohydrate intake, while the T and G carriers exhibit an improvement in insulin sensitivity, especially in the presence of low SFA consumption. Nonetheless, these results should be interpreted with caution, given the underrepresentation of ethnic groups other than those of European descent, the wide variety of dietary assessment, and the paucity of clinical studies, which would allow for an accurate control of macronutrient intake (Table 4).

Future interventional as well as large-scale observational prospective studies on populations of different ethnicities, such as those from Africa, Asia, and South America, are warranted to confirm the published findings and, possibly, to search for further relevant interactions between known loci and carbohydrate intake in modulating the development of T2D. In the meantime, experimental and clinical studies aimed at establishing which compounds in WGs are mainly involved in reducing the risk of T2D could provide valuable information for planning supplementation and nutritional programs, also based on the use of functional foods, in the framework of disease prevention and a personalized treatment strategy.

Considering that the strongest genetic variants account for approximately 20% of T2D heritability, epigenetics has proven to be decisive in explaining the missing heritability of T2D, in addition to rare variants. Differential methylation patterns, which determine a change in the expression of genes implicated in insulin release, energy balance, and glucose and lipid metabolism in blood and various tissues, have been shown to be crucial in modulating T2D risk. Hypermethylation at *CPT1A* cg00574958 is related to reduced MetS risk, decreased fasting triglycerides, and improved metabolic parameters. In contrast, a significant yet positive correlation between high fructose and carbohydrate consumption and *CPT1A*-cg00574958 methylation levels appears to profoundly affect the risk of metabolic traits, designating *CPT1A* as a mediator of the interaction between carbohydrates and T2D risk. However, despite the promising results, the current lack of longitudinal studies to confirm the provisional data and potentially identify novel biomarkers prevents the establishment of a causal relationship. A more complete picture of the methylome and the discovery of additional epigenetic markers mediating the effects of carbohydrate intake on T2D risk will provide a useful tool to predict individual disease risk and eventually plan appropriate nutritional treatments as adjuvants to traditional therapies.

## Figures and Tables

**Table 3:** Clues and pitfalls in the association between carbohydrate intake and risk of type 2 diabetes.

Clues	Reference	Pitfalls	Reference
High intake of carbohydrates significantly associated with an increased risk of T2D	[27]	Carbohydrate intake not associated with an increased risk of T2D	[162,163,168]
GI and GL significantly and positively associated with risk of T2D	[161,163,164,165]	No significant association of GI and GL with T2D incidence	[163]
No evidence of publication bias	[27,161,162]	Potential residual confounding	[27,161,162,169]
		Possibility of misclassification error and bias in the diagnosis and assessment of T2D (mostly based on self-reports)	[27,161,163,165,166]
		Most studies measure dietary intakes at baseline only	[27,161,163,165]
		Nutrition assessment used only FFQ and is therefore susceptible to large random and systemic errors	[27,161,162,163,164]
		Possibility of measurement errors in dietary assessment despite the improvement of methods	[169]
		Heterogeneity between studies due to differences in participant characteristics, geographical areas, and confounding factors	[27,161,164,165,166]
		No possibility to establish to what extent the effect of GL is attributable to carbohydrate intake	[161]
		Most studies conducted in female participants	[27]
		No causal relationship defined due to observational study design	[27,161,162,163,164,165]
		Publication bias between studies	[164,165,168]
		Possibility of misclassification in the assignment of GI and GL to food items	[166]
		Predominance of participants of European American descent	[164,169]

Abbreviations: FFQ; food frequency questionnaire; T2D: type 2 diabetes.

**Table 4:** Qualitative level of evidence on the interaction between carbohydrate intake and key genes for the risk of type 2 diabetes.

Carbohydrate Type	Gene	Variant	Effect of Interaction on T2D Risk	Level of Evidence
High intake of total fiber/cereal fiber/whole grains	*TCF7L2*	rs7903146 TT rs7903146 CC rs4506565 AA rs12255372	Harmful Protective Protective Harmful/Protective	Moderate Low Low Low
High intake of dietary fiber	*NOTCH2* *ZEBD2*	rs10923931 rs445705	Protective Protective	Low Low
High whole grain intake	*GCKR*	rs780094 C/CC	Harmful	Moderate
Low carbohydrate diet High carbohydrate intake Low short fatty acid -to-carbohydrate ratio	*IRS1*	rs2943641 T rs2943641 CC rs2943641 T rs7578326 G	Protective Protective Protective Protective	Low High Low Moderate

## Abbreviations

The following abbreviations are used in this manuscript:

BMI	Body mass index
CpG	Cytosine–guanine dinucleotides
CVD	Cardiovascular disease
DNMT	DNA methyltransferase
EWASs	Epigenome wide association studies
FFQ	Food frequency questionnaire
GCKR	Glucokinase regulator gene
GI	Glycemic index
GIP	Glucose dependent insulin polypeptide
GIPR	Glucose dependent insulin polypeptide receptor
GL	Glycemic load
GLP-1	Glucagon-like peptide-1
GWAS	Genome-wide association studies
HbA1c	Glycosylated hemoglobin A1c
HDL	High density lipoprotein
HOMA-IR	Homeostatic model assessment of insulin resistance
HR	Hazard ratio
IRS	Insulin receptor substrate
LCD	Low-carbohydrate diet
LDL	Low-density lipoprotein
LncRNA	Long non-coding RNA
MetS	Metabolic syndrome
miRNA	Micro-RNA
PPAR	Peroxisome proliferator-activated receptor
RCT	Randomized controlled trial
RR	Relative risk
SCFA	Short-chain fatty acid
SFA	Short fatty acid
SNP	Single-nucleotide polymorphisms
T2D	Type 2 diabetes
TCF7L2	Transcription factor 7-like 2
WGs	Whole grains
